# Optical Coherence Tomography-Based Positive Predictors of Eplerenone Therapy in Chronic Central Serous Chorioretinopathy: A Retrospective Study

**DOI:** 10.7759/cureus.58791

**Published:** 2024-04-22

**Authors:** Vijaya Sahu, Swatishree Nayak, Aseem Kumar, Himanshu Kashyap

**Affiliations:** 1 Ophthalmology, All India Institute of Medical Sciences, Raipur, IND; 2 Ophthalmology, Sankara Nethralaya, Chennai, IND

**Keywords:** double-layer sign, ophthalmology, subretinal fluid, central macular thickness, chronic central serous chorioretinopathy

## Abstract

Purpose: This study aimed to investigate optical coherence tomography (OCT) biomarkers as potential predictors of treatment response in chronic central serous chorioretinopathy (CSCR).

Materials and methods: It was a retrospective cohort study that included 42 patients with chronic CSCR. After complete ocular and hematological examinations, all patients received 50 mg/day of oral eplerenone for three months and were followed for at least six months. All participants were divided into two groups: Group 1 participants with a positive response to treatment (complete resolution of subretinal fluid (SRF) at six months) and Group 2 poor responders (moderate or less than 50% reduction in SRF from baseline). The primary outcome measure was the resolution of SRF, and various OCT biomarkers like central macular thickness (CMT), pigment epithelial detachments (PED), double-layer sign, elongation of the photoreceptor's outer segment, the integrity of the external limiting membrane, the integrity of the ellipsoid zone, hyperreflective foci in the outer segment, and subretinal deposits in the SRF were assessed.

Results: The mean age was 41.33 ± 10.75 years, and 34 participants were male. Thirty-seven (88.1%) of the participants had good responses to eplerenone, with the mean height of SRF decreasing significantly from a maximum of 269.74 µm to a minimum of 21.86 µm at six months (p<0.001). The mean CMT decreased from the first visit time point to the third visit time (p<0.001). Logistic regression analysis assessed the absence of PED and double-layer signs associated with a good response.

Conclusion: The eplerenone therapy seems to be efficient for chronic CSCR, and OCT can be an invaluable aid to the treating physician.

## Introduction

Central serous chorioretinopathy (CSCR) stands out as a prevalent condition encountered in medical retinal practice, characterized by serous retinal detachment with or without retinal pigment epithelial detachment (PED) [1]. Despite an unclear underlying pathophysiology, it is hypothesized to involve fluid leakage through the retinal pigment epithelium (RPE) into the subretinal space. Commonly affecting young adult males, CSCR ranks as the fourth most common cause of medical retinopathy, following age-related macular degeneration, retinal venous occlusion, and diabetic retinopathy, with an incidence of approximately 5.8 per 100,000 people [2].

Acute CSCR typically presents as a benign disorder that resolves spontaneously within 2-3 months. However, around 5-10% of cases may progress to the chronic form when fluid persists for more than three months, presenting as diffuse retinal epitheliopathy. This chronic manifestation is characterized by persistent or recurrent detachments of neuroretina, diffuse RPE alterations, cystoid retinal degeneration, or pigment epithelium detachment [3]. Recurrent chronic CSCR affects 30-50% of patients [4,5]. In chronic cases, persistent fluid accumulation can lead to progressive and irreversible damage to photoreceptors, significantly impacting the visual prognosis [6-8]. Animal research has suggested that activation of choroidal mineralocorticoid receptors induces choroidal vasodilation and leakage, contributing to CSCR. Antagonism of the mineralocorticoid receptor has shown preventive effects in this context [9]. Spironolactone and eplerenone, both oral-specific mineralocorticoid receptor antagonists, are being tested for use in the medical management of CSCR. Eplerenone, a selective aldosterone-receptor antagonist and potassium-sparing diuretic initially approved by the Food and Drug Administration (FDA) in 2002 for hypertension and later in 2003 for congestive heart failure after myocardial infarction, has demonstrated efficacy in improving visual acuity in CSCR cases [10]. It has also shown a significant decrease in central macular thickness (CMT) and subfoveal fluid [8,9].

These key parameters can be accurately monitored using optical coherence tomography (OCT), which provides high-resolution, cross-sectional images of the retina, allowing for detailed visualization of retinal layers, fluid accumulation, and structural changes. Several authors have successfully utilized OCT-based biomarkers over the years to better understand the condition and predict treatment outcomes [11,12].

In addition to assessing the overall efficacy of eplerenone over six months, this study aims to investigate OCT biomarkers as potential predictors of treatment response in chronic CSCR cases. Identifying reliable predictors could facilitate a more targeted and personalized approach to the management of CSCR, thereby improving overall treatment outcomes and patient care.

## Materials and methods

Study design

This retrospective cohort study was conducted at the All India Institute of Medical Sciences, Raipur, India. Recruitment of patients was done from June 1 to December 31, 2021. The study protocol was approved by the Institute Ethics Committee of All India Institute of Medical Sciences (approval number: AIIMSRPR/IEC/2022/1273), and the tenets of the Declaration of Helsinki were strictly adhered to throughout the study. Written informed consent was obtained from each participant. The study met all the criteria of the Strengthening the Reporting of Observational Studies in Epidemiology (STROBE) Statement checklist.

Participants

Out of 51 patients assessed, 42 eligible patients were enrolled. Patients with chronic CSCR identified through clinical examination and the presence of subretinal fluid (SRF) involving the fovea on OCT for more than three months were included in the study. Fundus fluorescence angiography was done in all patients. The exclusion criteria were patients below 18 years of age, those who were pregnant or planning to conceive, and those who had a history of liver or kidney disease, hyperkalemia (>5.0 mmol/L), and high serum creatinine level (>2 mg/dL in males, >1.8 mg/dL in females). Patients with any evidence of choroidal neovascularization, with a history of other retinal abnormalities like diabetes, hypertension, or chronic uveitis that cause macular changes, and who received photodynamic therapy (PDT), anti-vascular endothelial growth factor (anti-VEGF), or laser treatment were also excluded from the study.

Sample size calculation

The sample size was calculated by taking the confidence interval as 95% and the power as 80%. The proportion of subjects with resolution was taken as 58.3%, as reported by a study by Kapoor and Wagner [13]. Using the appropriate formula, the sample size was found to be 42.

Intervention

Every participant underwent a comprehensive ocular examination and hematological investigations for serum potassium level and kidney function before treatment, with monthly repetitions. All patients received 50 mg/day of oral eplerenone for three months and were followed for at least six months. The chosen dose (50 mg/day) is the lowest efficient concentration in many studies with high specificity. A minimum of three spectral domain OCT imaging using the Zeiss Cirrus 5000 device with high-resolution OCT-21 HD scans centered on the fovea and macular cube 512 x 128 or 200 x 200 scans were performed to ensure each case had a baseline (the first visit at the end of the one-month treatment, the second visit at the end of the three-month treatment, and the third visit at the end of the six-month follow-up).

Outcomes

The primary outcome measure was the resolution of SRF. All study participants with chronic CSCR who were started on eplerenone were categorized into two groups depending on treatment response: Group 1, positive response to treatment (complete resolution of SRF at six months), and Group 2, poor responders (moderate or less than 50% reduction in SRF from baseline). The other OCT biomarkers like CMT, subfoveal and extrafoveal PED, double-layer sign, elongation of photoreceptors' outer segment, integrity of the external limiting membrane, integrity of the ellipsoid zone, hyperreflective foci in the outer segment, and subretinal deposits in the SRF were noted in both groups.

Statistical methods

Categorical variables like gender, resolution or non-resolution of SRF, and presence or absence of OCT biomarkers were presented as percentages and analyzed by the chi-squared test. Continuous variables like age, change in the height and width of SRF over time, and change in CMT were described as mean ± standard deviation. The Shapiro-Wilk test was used to check for the normality of the data. The comparison between the baseline and follow-up measurements was done by the Wilcoxon signed-rank test when the data was found to have a non-normal distribution. The Spearman correlation test was used to assess the correlation between OCT parameters and SRF resolution. Clinical and OCT parameters at baseline were correlated with response to treatment at three and six months of follow-up using logistic regression analysis. A p-value of <0.05 was considered significant. All analysis was performed using IBM SPSS Statistics for Windows, V. 24.0 (IBM Corp., Armonk, NY).

## Results

Basic information

Fifty-one patients were assessed for eligibility. Nine patients were excluded, and the remaining 42 patients were recruited for the study. Overall, 42 eyes of 42 patients (mean age 41.33 ± 10.75 years, 34 males) were included in the analysis. When the study population was stratified according to age groups, the maximum number of patients was found to be in the age group of 31-40 years (Table 1).

**Table 1:** Demographic details of patients recruited IQR: interquartile range

Variables	Mean ± SD	Median (IQR)	Min-max	Frequency (%)
Age (years)	41.33 ± 10.75	39.50 (34.00-44.75)	24.00-72.00	
21-30				2 (4.8%)
31-40				22 (52.4%)
41-50				10 (23.8%)
51-60				5 (11.9%)
61-70				2 (4.8%)
71-80				1 (2.4%)
Gender				
Male				34 (81.0%)
Female				8 (19.0%)

At six-month follow-up, 37 (88.1%) patients were found to have a positive response to treatment with eplerenone, while five (11.9%) were poor responders. The qualitative analysis of different OCT biomarkers, along with relevant etiological factors, was evaluated. Disturbed sleep patterns were the most common etiological factor, while hyperreflective dots were the most commonly seen observation on OCT (Table 2).

**Table 2:** Summary of features PED: pigment epithelial detachments

Features	Yes, N(%)	No, N(%)
Fibrin	3 (7.1%)	39 (92.9%)
PED	12 (28.6%)	30 (71.4%)
Cystoid changes	3 (7.1%)	39 (92.9%)
Double-layer sign	15 (35.7%)	27 (64.3%)
Hyperreflective dots	23 (54.8%)	19 (45.2%)
Steroid use	5 (11.9%)	37 (88.1%)
Fairness cream use	8 (19.0%)	34 (81.0%)
Disturbed sleep pattern	21 (50.0%)	21 (50.0%)

**Figure 1:**
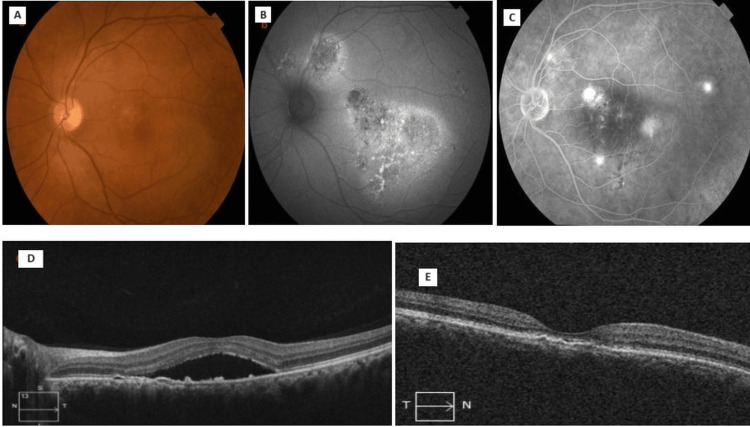
Evaluation of the status a) Fundus photo of a patient showing chronic CSCR with areas of RPE mottling. b) Fundus autofluorescence image illustrating a mixed hyper- and hypoautofluorescence intensity over the area of RPE mottling. c) Fundus fluorescein angiography showing multifocal leakage of dye and diffuse RPE defect resulting in patches of granular or mottled hyperfluorescence. d) OCT scan showing subretinal fluid at the first visit. e) OCT scan showing resolution of subretinal fluid at six months after eplerenone therapy (Group 1: positive response to treatment) CSCR: central serous chorioretinopathy; RPE: retinal pigment epithelium; OCT: optical coherence tomography

Thirty-seven (88.1%) of the participants had good responses to eplerenone in terms of a decrease in the height of SRF and CMT (Figure 1).

The first visit was at the end of the one-month treatment, the second visit at the end of the three-month treatment, and the third visit at the end of the six-month follow-up. While none of the patients showed resolution of SRF at the first visit, the overall change in resolution of SRF at the end of three months (n = 22 (52.4%); McNemar's test: χ^2^ = 22.000, p<0.001) and six months (n = 37 (88.1%); McNemar's test: χ^2^ = 37.000, p<0.001) were statistically significant (Table 3).

**Table 3:** Summary of resolution of SRF The p-value was considered significant at <0.05 SRF: subretinal fluid

Resolution of SRF	Yes, N(%)	No, N(%)
First visit	0 (0.0%)	42 (100.0%)
Second visit	22 (52.4%)	20 (47.6%)
Third visit	37 (88.1%)	5 (11.9%)

**Figure 2:**
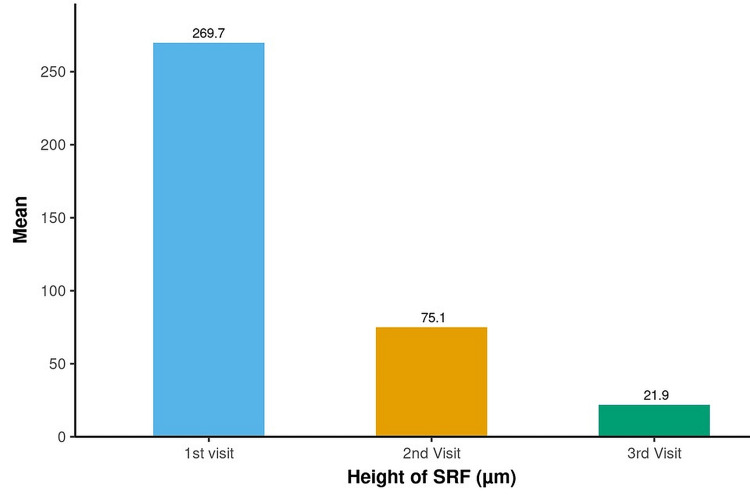
Assessment of change in height of SRF (µm) over time The p-value was considered significant at <0.05 SRF: subretinal fluid

On analyzing the OCT parameters, the mean height of SRF (fovea is taken as the point for measurement) decreased significantly from a maximum of 269.74 µm to a minimum of 21.86 µm at six months (Figure 2).

**Figure 3:**
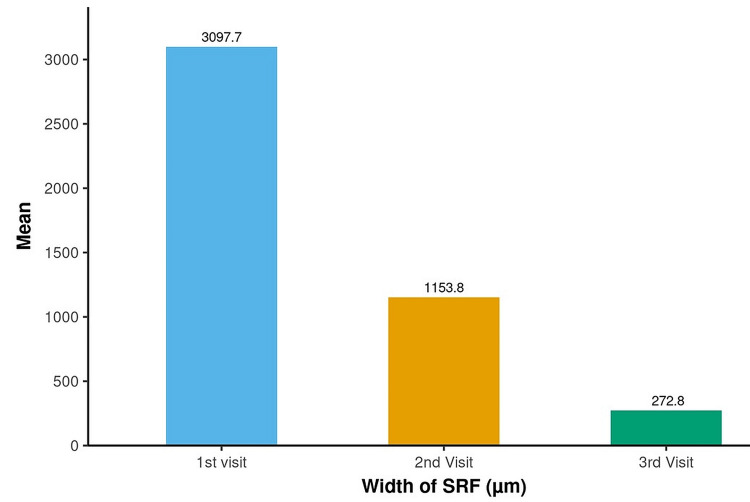
Assessment of change in width of SRF (µm) over time The p-value was considered significant at <0.05 SRF: subretinal fluid

The observed change in the mean width of SRF from 3097.71 µm to 272.76 µm at the third visit was statistically significant (p<0.001) (Figure 3).

**Figure 4:**
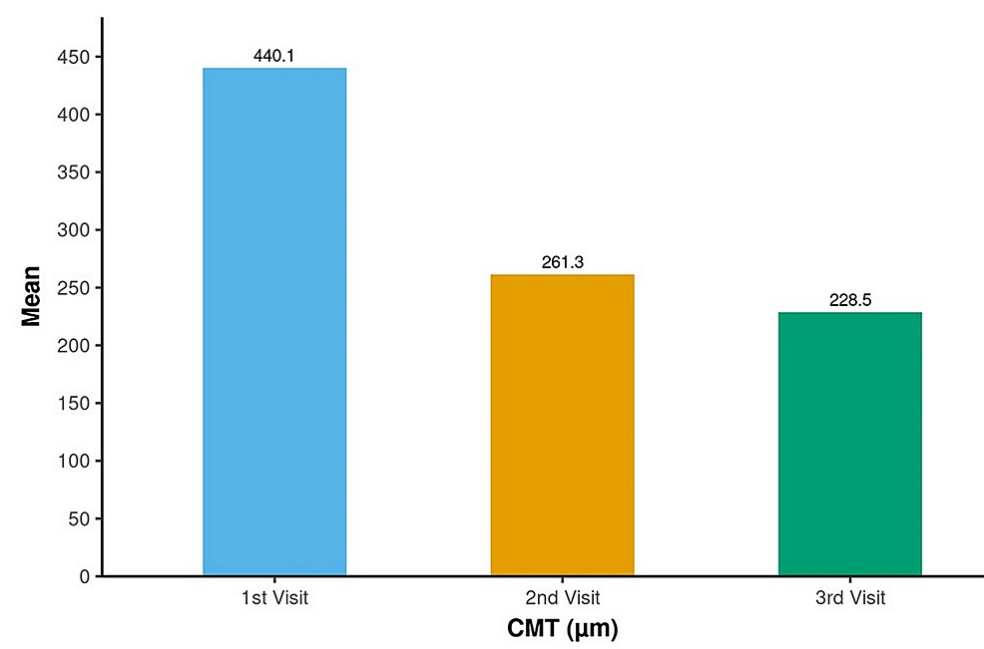
Assessment of change in CMT (µm) over time The p-value was considered significant at <0.05 CMT: central macular thickness

The mean CMT decreased from a maximum of 440.12 µm at the first visit time point to a minimum of 228.52 µm at the third visit time point, which was statistically significant (p<0.001) (Figure 4).

There was no significant difference between the two groups in terms of age (p = 0.341). The point-biserial correlation indicated a small effect size of 0.16. There was no significant difference between the two groups in terms of the distribution of fibrin, PED, cytoid changes, double-layer sign, and hyperreflective dots. There was a significant difference between the two groups in terms of the height of SRF at the first, second, and third visits (W = 35.000, p = 0.023). The strength of the association indicated a medium to large effect size. A large effect size of 0.55 was noted in the width of SRF between the two groups in the second and third visits. Despite some observed changes in visual acuity, the differences were not statistically significant between the two groups. The width was measured with the caliper by line drawing between side-to-side end points of neurosensory detachment, and the resolution was measured as the presence or absence of SRF (Table 4).

**Table 4:** Association between resolution of SRF and OCT parameters The p-value was considered significant at <0.05 PED: pigment epithelial detachments; CMT: central macular thickness; SRF: subretinal fluid; OCT: optical coherence tomography

Parameters	Response of SRF		P-value
	Good (n = 37)	Poor (n = 5)	
Fibrin (yes)	3 (8.1%)	0 (0.0%)	1.000
PED (yes)	10 (27.0%)	2 (40.0%)	0.613
Cystoid changes (yes)	3 (8.1%)	0 (0.0%)	1.000
Double-layer sign (yes)	12 (32.4%)	3 (60.0%)	0.329
Hyperreflective dots (yes)	21 (56.8%)	2 (40.0%)	0.644
Steroid use (yes)	5 (13.5%)	0 (0.0%)	1.000
Fairness cream use (yes)	5 (13.5%)	3 (60.0%)	0.040
Disturbed sleep pattern (yes)	20 (54.1%)	1 (20.0%)	0.343
Height of SRF (µm) (first visit)	246.76 ± 225.00	439.80 ± 192.73	0.023
Width of SRF (µm) (first visit)	2965.30 ± 1866.77	4077.60 ± 850.30	0.151
Height of SRF (µm) (second visit)	58.62 ± 92.33	196.80 ± 97.99	0.003
Width of SRF (µm) (second visit)	869.38 ± 1199.10	3258.80 ± 1242.91	0.002
Height of SRF (µm) (third visit)	0.00 ± 0.00	183.60 ± 108.45	<0.001
Width of SRF (µm) (third visit)	0.00 ± 0.00	2291.20 ± 312.90	<0.001
Resolution of SRF (first visit) (yes)	0 (0.0%)	0 (0.0%)	1.000
Resolution of SRF (second visit) (yes)	22 (59.5%)	0 (0.0%)	0.018
Resolution of SRF (third visit) (yes)	37 (100.0%)	0 (0.0%)	<0.001
CMT (µm) (first visit)	425.32 ± 188.66	549.60 ± 177.24	0.125
CMT (µm) (second visit)	255.86 ± 61.31	301.60 ± 12.22	0.036
CMT (µm) (third visit)	219.03 ± 22.81	298.80 ± 103.96	0.017

Logistic regression analysis was performed to assess the predictors for complete resolution of macular SRF at six months. The absence of PED and double-layer signs seems to be associated with a higher likelihood of macular SRF resolution, while higher height and width of SRF are associated with a lower likelihood (Table 5).

**Table 5:** Association between resolution of SRF and various predictors PED: pigment epithelial detachments; SRF: subretinal fluid

Predictor/risk factor	Outcome	Odds ratio (95% CI)	Relative risk (95% CI)
Fibrin: yes	Resolution of SRF (third visit): yes	1.12 (0.05-24.69)	1.15 (0.5-1.36)
PED: yes	Resolution of SRF (third visit): yes	0.56 (0.08-3.83)	0.93 (0.61-1.18)
Cystoid changes: yes	Resolution of SRF (third visit): yes	1.12 (0.05-24.69)	1.15 (0.5-1.36)
Double-layer sign: yes	Resolution of SRF (third visit): yes	0.32 (0.05-2.18)	0.86 (0.59-1.1)
Hyperreflective dots: yes	Resolution of SRF (third visit): yes	1.97 (0.29-13.21)	1.08 (0.84-1.48)

**Figure 5:**
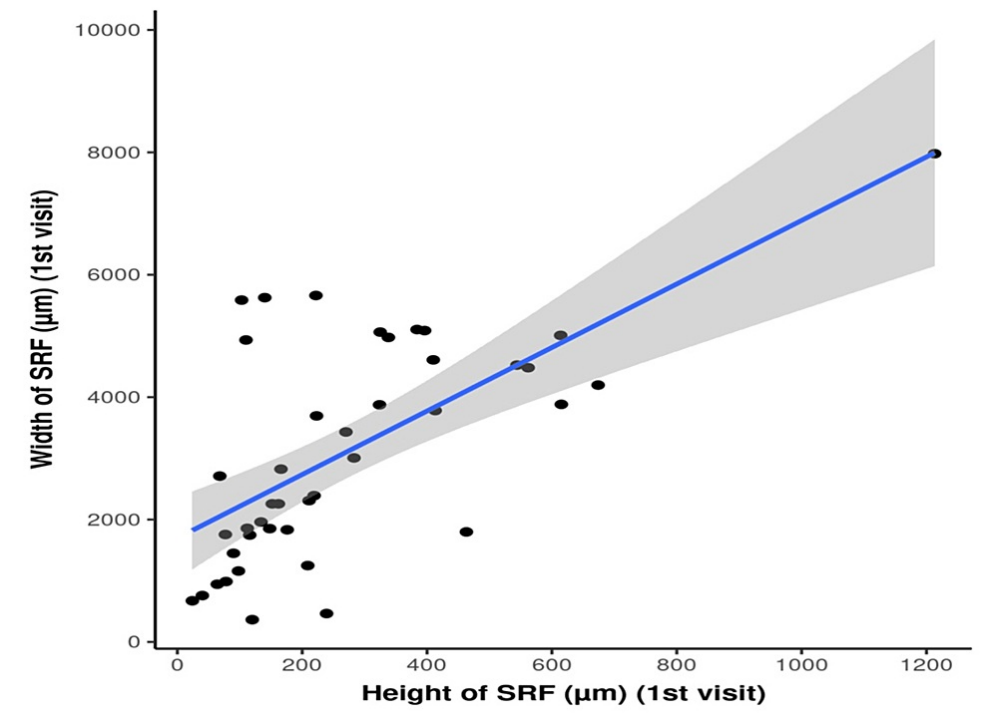
Scatter plot showing the correlation between the height and width of SRF The p-value was considered significant at <0.05 SRF: subretinal fluid

There was a strong positive correlation between the width and height of SRF at the first visit (rho = 0.6, p = 0.001). For every 1 unit increase in the width of SRF at the first visit, the height of SRF increases by 0.08 units. Conversely, for every 1 unit increase in the height of SRF at the first visit, the width of SRF increases by 5.19 units (Figure 5).

**Figure 6:**
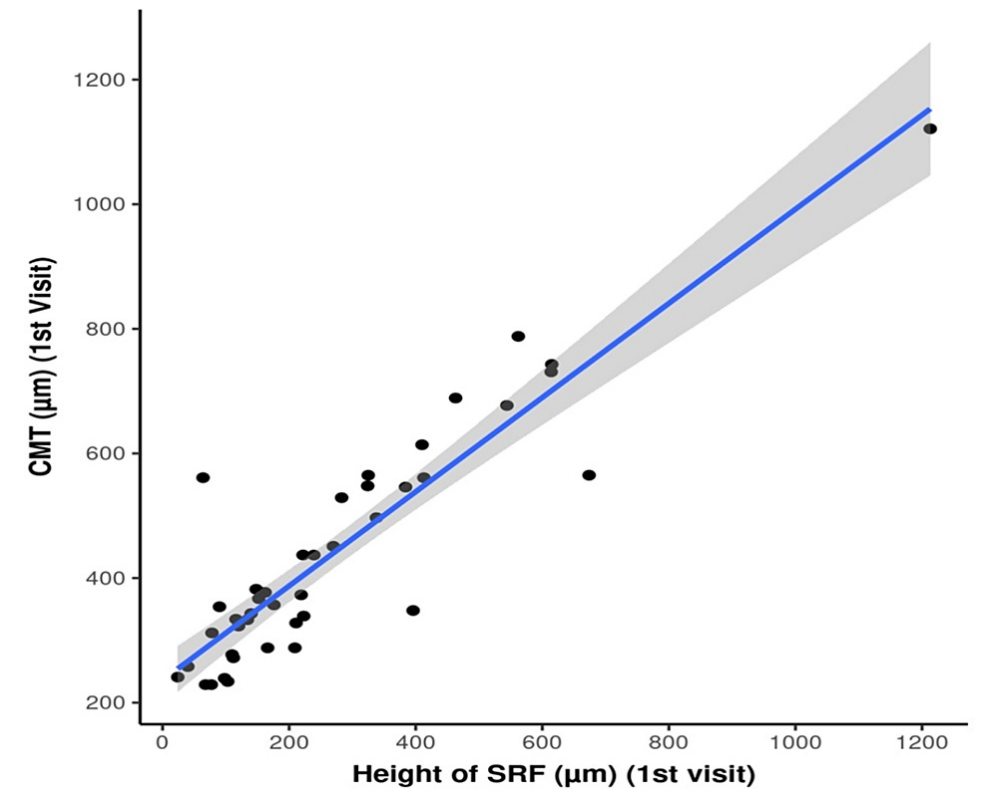
Scatter plot showing the correlation between the height of SRF and CMT The p-value was considered significant at <0.05 SRF: subretinal fluid; CMT: central macular thickness

There was a strong positive correlation between the height of SRF and CMT at the first visit (rho = 0.84, p<0.001). For every 1 unit increase in height of SRF, the CMT increases by 0.76 units. Conversely, for every 1 unit increase in CMT, the height of SRF increases by 1.09 units (Figure 6).

None of the patients complained of side effects during the evaluation period. No abnormalities in electrolytes, ECG, or blood pressure were noted in the six months duration.

## Discussion

This retrospective cohort study focuses on the functional and anatomical response to eplerenone over six months in individuals with chronic CSCR. The study suggests that eplerenone treatment is effective in these cases by reversing choroidal vasodilatation, leading to the resolution of SRF. Aldosterone induces the upregulation of endothelial vasodilatory potassium channel KCa2.3, a calcium-dependent channel crucial for endothelial hyperpolarization and vasorelaxation. The reversal of upregulation and activation of this channel-induced vasodilation in the choroid is evident with mineralocorticoid receptor antagonism by eplerenone. The initial positive findings align with a study by Zhao et al., where two chronic CSCR patients treated with oral eplerenone experienced rapid resolution of SRF and improved visual acuity, maintained even after the cessation of treatment [9]. Following this, other studies investigated the efficacy and safety of eplerenone for CSCR treatment, with the present study yielding similar positive results [10-16].

However, it's important to note that not all clinical results have been uniformly convincing. A well-designed, double-blinded, placebo-controlled trial (VICI) has reported that eplerenone was not found to be superior to placebo for chronic CSCR [17]. This contrasts with positive outcomes reported in earlier studies and highlights the complexity of treating chronic CSCR, with differing results across various investigations. It's worth considering the potential reasons for these discrepancies, such as variations in patient populations, study designs, and the multifactorial nature of CSCR.

Our study provides valuable insights into the effects of eplerenone treatment on CSCR by investigating various biomarkers and outcomes. In our study, a significant decrease in SRF was noted just after one month of eplerenone treatment. Notably, there are conflicting findings in the literature, with some studies reporting a significant SRF reduction after one month, while others, like Bousquet et al., did not find a significant change [10,18,19]. They observed complete reabsorption of SRF only in 25% of patients at one month and in 67% of patients by the end of three months.

CMT decreased significantly after four weeks of eplerenone treatment in our study, consistent with findings in other studies [10,20-22]. However, there are discrepancies as Schwartz et al. did not find the CMT changes superior in the eplerenone group as compared to placebo [18]. Another study by Ghadiali et al., which involved long-term follow-up of CSCR patients treated with mineralocorticoid antagonists, reported a decrease in SRF but did not observe a significant change in CMT and choroidal thickness [23]. It is noteworthy that both these studies included patients who had undergone PDT, received intravitreal anti-VEGF injections, and underwent macular laser which might have influenced the results.

In the present study, best corrected visual acuity (BCVA) did not change significantly after one month of eplerenone treatment, and there was no significant correlation between SRF height or CMT and visual acuity. Similar results were reported by Schwartz et al. and Bousquet et al., where no clinically significant improvement in BCVA was seen after 30 days of spironolactone treatment [10,18]. However, Rahimy et al. and Daruich et al. noted a significant change in BCVA after six months of treatment [11,20]. This reinforces the idea that using fluid resolution as a biomarker for positive responses might be more prudent than relying solely on visual acuity. Visual acuity can be influenced by various factors, including disruptions in retinal layers like the ellipsoid zone, the external limiting membrane, hyperreflective foci in retinal layers, and the severity of RPE atrophy, rather than being solely dependent on the resolution of SRF.

There is considerable variability in the designs of studies related to eplerenone therapy for CSCR. While our study specifically addressed the efficacy of eplerenone therapy in chronic CSCR patients, some studies compared eplerenone therapy to observation alone in cases of acute CSCR [24]. Other studies assessed efficacy across both acute and chronic CSCR. Our findings highlight factors associated with a poorer response to eplerenone, such as long SRF height, wide SRF diameter, the presence of PED, and the double-layer sign. Borrelli et al. reported a wider SRF diameter and an increased number of serous PED associated with a poorer response [25]. They suggested that advanced RPE epitheliopathy at baseline and consequently diminished RPE cell ability to reabsorb SRF during eplerenone treatment may contribute to these outcomes. Notably, our study differed from theirs concerning hyperreflective foci as indicators of poor treatment response. The inclusion of acute CSCR patients in their study could be a contributing factor to this divergence.

The study's main constraint was its limited sample size. Conducting a larger multicentric study could have facilitated the generalization of results. A randomized controlled trial would have offered a more robust study design. Nevertheless, introducing a control group of chronic CSCR patients without any treatment would have presented ethical challenges.

## Conclusions

Therapy with eplerenone seems to be efficient at the chronic stage of the disease. We believe that a definition of predictive biomarkers is useful to identify patients who most likely can benefit from treatment. Overall, our study contributes to the ongoing dialogue surrounding eplerenone therapy for CSCR, emphasizing the need for comprehensive and nuanced approaches to understanding and managing this complex retinal disorder.
